# Nedaplatin: A Radiosensitizing Agent for Patients with Cervical Cancer

**DOI:** 10.1155/2011/963159

**Published:** 2010-09-21

**Authors:** Seiji Mabuchi, Tadashi Kimura

**Affiliations:** Department of Obstetrics and Gynecology, Osaka University Graduate School of Medicine, 2-2 Yamadaoka, Suita, Osaka 565-0871, Japan

## Abstract

Despite the recent advances in the management of cervical cancer using cisplatin-based concurrent chemoradiotherapy, substantial treatment failure still occurs, especially in advanced-stage patients and early-stage cervical cancer patients with high-risk prognostic factors. Therefore, efforts to further improve the survival and quality of life of these patients are necessary. Nedaplatin (cis-diammine-glycoplatinum), a derivative of cisplatin, was developed with the aim of producing a treatment with a similar effectiveness to cisplatin but decreased renal and gastrointestinal toxicities. Based on the promising results of preclinical studies, the clinical efficacy of nedaplatin as a radiosensitizing agent was evaluated in patients with cervical cancer. Retrospective analysis of nedaplatin-based concurrent chemoradiotherapy (CCRT) against cervical cancer suggested that nedaplatin-based CCRT can be considered as an alternative to cisplatin-based CCRT in both early-stage and advanced-stage cervical cancer patients. However, due to the lack of a randomized controlled study, nedaplatin-based CCRT has not been convincingly proven to be clinically effective in patients with cervical cancer. Further investigations in randomized controlled trials are therefore needed.

## 1. Introduction

**Figure 1:**
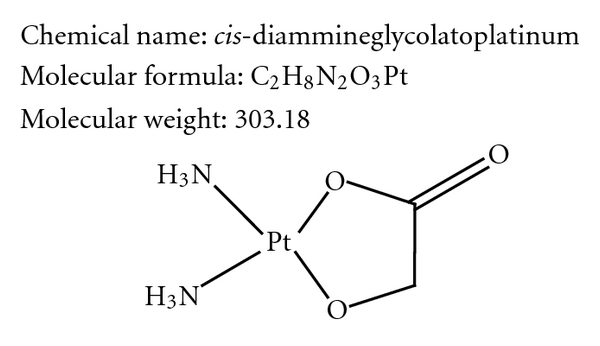
Chemical structure of nedaplatin.

Nedaplatin (cis-diammine-glycoplatinum), a derivative of cisplatin, was developed in 1983 by Shionogi Pharmaceutical Company in Japan with the aim of producing a treatment with a similar effectiveness to cisplatin but decreased renal and gastrointestinal toxicities [1]. As shown in Figure 1, nedaplatin has a novel chemical structure involving a five-membered ring structure in which glycolate is bound to the platinum ion as a bidentate ligand.

 In a preclinical evaluation of cervical cancer, nedaplatin demonstrated similar antitumor activity to cisplatin [2, 3]. Its lower incidence of nephrotoxicity in comparison to cisplatin has been demonstrated to be associated with differences in the kidney distribution of these drugs. When the two agents were administered at the same dose, the accumulation of nedaplatin in the rat kidney was approximately 40% of that of cisplatin, which explains why nedaplatin is associated with less nephrotoxicity than cisplatin [4, 5].

 Clinically, previous phase II studies conducted in Japan suggested that nedaplatin has a particularly favorable clinical efficacy towards squamous cell carcinoma (SCC) of the lung, head and neck, esophagus, and uterine cervix [6–9]. According to a clinical study of nedaplatin in nonsmall cell lung cancer patients, the response rate in patients with SCC histology was 57.1%, which is significantly higher than 5.5% observed in patients with nonSCC histology [9]. In the area of uterine cervical cancer, in a phase II clinical trial, nedaplatin demonstrated a response rate of 46% in patients with recurrent cervical cancer, which was slightly superior to that obtained with cisplatin (39%) [8]. Moreover, since nedaplatin does not require hydration, nedaplatin treatment can be managed in an outpatient setting. On the basis of these advantages, nedaplatin has been used clinically in Japan as an alternative to cisplatin for patients with recurrent cervical cancer [10].

 The radiosensitizing properties of nedaplatin have been demonstrated in several preclinical studies [11, 12]. An in vitro investigation demonstrated that nedaplatin in combination with irradiation is highly effective for cervical cancer [12]. Although the preliminary data from clinical studies of the use of nedaplatin-based CCRT in patients with head and neck or esophageal cancer has been reported [13], however, the clinical experience with this agent in the setting of CCRT for cervical cancer patients is limited. As shown in Table 1, the use of concurrent weekly nedaplatin in patients with invasive cervical cancer in the setting of definitive radiotherapy was investigated in two Phase I [14, 15], two Phase II [16, 17] studies, and one retrospective study [18]; however, in the setting of adjuvant radiotherapy, nedaplatin-based CCRT has only been evaluated in one Phase I [19] and two retrospective studies [20, 21]. Thus, it remains unclear whether nedaplatin-based CCRT is superior to RT alone in patients with cervical cancer.

 Concurrent chemoradiotherapy, usually involving 40 mg/m^2^ of weekly cisplatin, is accepted as the standard first-line treatment for cervical cancer [32, 33]. However, its nephrotoxicity and gastrointestinal toxicity may limit its use. In a previous Japanese Phase I study, which determined the recommended dose of weekly cisplatin in the setting of CCRT after radical hysterectomy, dose-limiting toxicity (DLT) was observed at 40 mg/m^2^, indicating that a weekly dose of 40 mg/m^2^ of cisplatin may be too high for Japanese patients with cervical cancer [34]. As nedaplatin exhibits minimal nephrotoxicity, it can be used in patients with marginal renal function [4, 5]. Moreover, since nedaplatin does not require hydration and shows minimal gastrointestinal toxicity, nedaplatin treatment can be managed in an outpatient setting. Thus, the substitution of nedaplatin for cisplatin as a concurrent chemotherapy for patients with cervical cancer may be beneficial. 

Recently, we investigated the efficacy of nedaplatin-based CCRT in the settings of adjuvant treatment after radical hysterectomy [18] and definitive radiotherapy [20]. Although they were retrospective in nature, to the best of our knowledge, these are the only reports that have demonstrated a significant improvement in the survival of cervical cancer patients treated with nedaplatin-based CCRT. In this review, we show the results of these retrospective analyses and provide information on the results of other clinical studies that investigated the efficacy of nedaplatin-based CCRT in patients with cervical cancer.

## 2. Postoperative Concurrent Nedaplatin-Based Chemoradiotherapy in Patients with Early-Stage Cervical Cancer

### 2.1. Background

Early-stage cervical cancer has traditionally been treated with either radical hysterectomy or primary radiotherapy, with similar survival outcomes [35]. Several risk factors have been identified that compromised the treatment outcome in patients with Early-stage cervical cancer who were primarily treated with radical surgery [36–38]. Generally, patients that demonstrate risk factors such as positive pelvic nodes, parametrial invasion, and a positive vaginal margin are regarded as “high-risk” for recurrence [36]. Moreover, patients with a tumor confined to the cervix that display risk factors such as a large tumor, lymph vascular space invasion, and deep stromal invasion are considered to be at “intermediate-risk” of recurrence [37, 38]. Postoperative RT is usually recommended for patients that display these risk factors.

 A previous Gynecologic Oncology Group Phase III study (GOG 92) evaluated the role of adjuvant RT in patients that showed “intermediate-risk” prognostic factors, that is, those with at least two of the following risk factors after radical hysterectomy: >1/3 stromal invasion, lymph vascular space involvement, or a large tumor diameter. Although overall survival was not significantly prolonged by the addition of adjuvant RT, this study demonstrated that adjuvant RT significantly reduced the risk of recurrence and prolonged progression-free survival in these women [39, 40]. However, the clinical benefit of postoperative CCRT in patients with Early-stage cervical cancer that displayed intermediate-risk prognostic factors has never been investigated in the setting of randomized clinical trials.

In early-stage cervical cancer patients demonstrating high-risk prognostic factors, a prospective randomized clinical trial (GOG 109/SWOG 87-97) examined the role of adjuvant cisplatin-based CCRT after radical hysterectomy and pelvic lymphadenectomy [41]. The study demonstrated that the addition of concurrent cisplatin-based chemotherapy to postoperative RT improved the survival of patients with positive pelvic LN and/or a positive resection margin and/or parametrial involvement. 

Thus, it remains unclear whether postoperative nedaplatin-based CCRT is superior to RT alone in patients with Early-stage cervical cancer that displayed intermediate- or high-risk prognostic factors.

To answer this question, we retrospectively evaluated the effectiveness of nedaplatin-based CCRT in 145 Japanese patients with FIGO Stage IA2-IIB cervical cancer after radical hysterectomy and pelvic lymphadenectomy [18].

### 2.2. Clinical Findings

As shown in Tables 2 and 3, among the patients enrolled in the study, 57 showed intermediate-risk prognostic factors such as deep stromal invasion, capillary lymphatic space involvement, or a large tumor diameter. Sixty-eight patients displayed high-risk prognostic factors such as positive pelvic lymph nodes, parametrial involvement, or a positive surgical margin. These patients were postoperatively treated with either CCRT or RT alone.

 The optimal dose of concurrent weekly nedaplatin has not been well established in the setting of adjuvant therapy because nedaplatin-based adjuvant CCRT has only been prospectively evaluated in one Phase I study [19], in which the authors recommended 35 mg/m^2^ nedaplatin for 5 weeks as a standard treatment regimen (Table 1). However, since weekly 40 mg/m^2^ of cisplatin in combination with pelvic radiotherapy has been established as a standard treatment for cervical cancer, moreover, since the antitumor activity of nedaplatin has been reported to be similar to that of cisplatin [2, 3, 8], we have employed 40 mg/m^2^ of weekly nedaplatin for 5 weeks as a standard regimen for adjuvant CCRT in our clinical practice.

Overall, nedaplatin-based CCRT was well tolerated. There were no treatment-related deaths, and all patients completed the planned pelvic RT. As shown in Table 4, among a total of 56 patients treated with CCRT, grade 3 or 4 acute toxicities were observed in 36 patients (64.3%). Of these, thirty patients had neutropenia alone and three had both neutropenia and thrombocytopenia. Three patients developed nonhematologic toxicities (bowel obstruction in one patient, diarrhea in one patient, radiation dermatitis in one patient). Among a total of 69 patients treated with RT alone, grade 3 or 4 acute toxicities were observed in 8 patients (11.6%). Of these, four patients had neutropenia alone and one had both neutropenia and thrombocytopenia. Three patients developed nonhematologic toxicities (bowel obstruction in one patient, diarrhea in two patients.). When compared, the frequency of acute grade 3-4 acute toxicities observed in patients treated with CCRT was significantly higher than that observed in the RT-group (*P* < .001). Nevertheless, there were no significant differences in the length of radiotherapy among these treatment groups (Table 2 and 3). In this study, severe late complications were only observed in a patient treated with RT (Table 4). This woman developed a vesicovaginal fistula 4 months after the completion of RT. No Grade 3-4 late toxicity was observed in the patients treated with CCRT, which may indicate that the addition of concurrent weekly nedaplatin to pelvic RT does not increase late toxicity.

**Figure 2:**
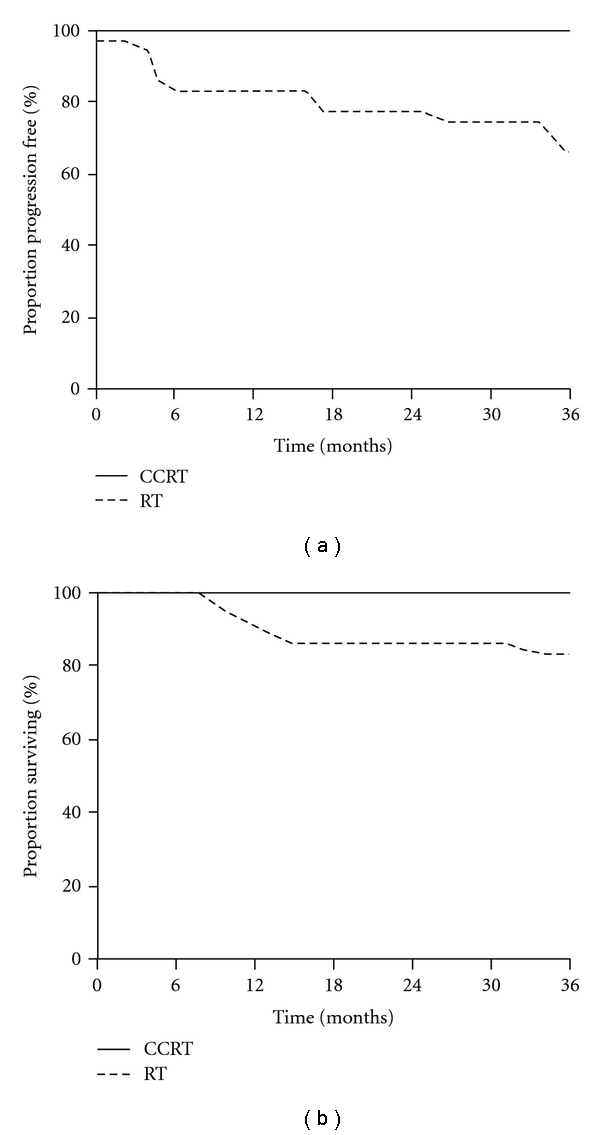
Progression-free survival (a) and overall survival (b) in intermediate-risk patients.

**Figure 3:**
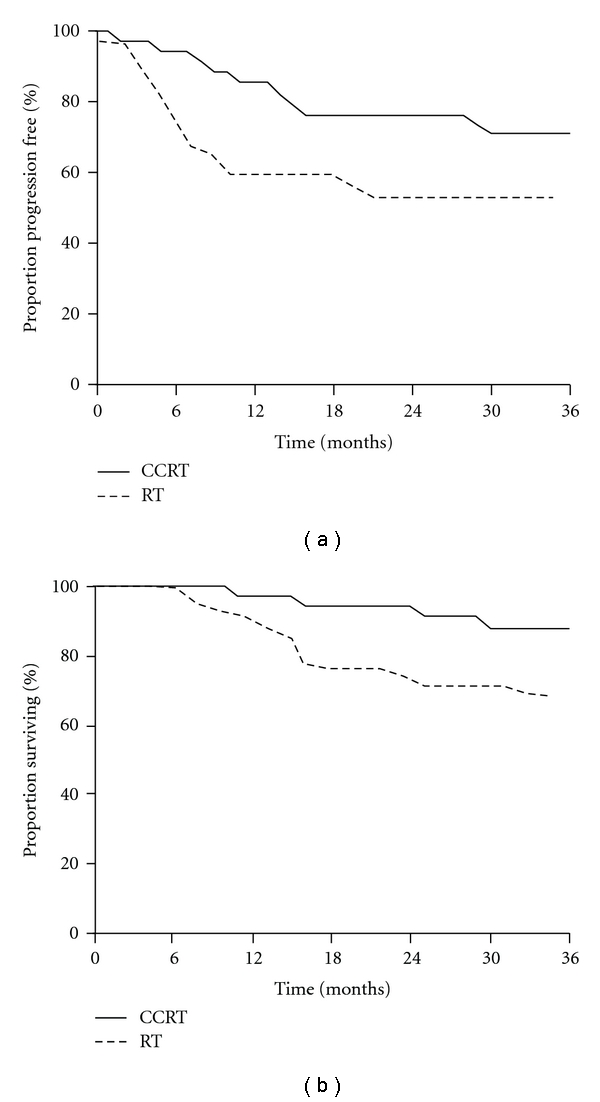
Progression-free survival (a) and overall survival (b) in high-risk patients.

 In the intermediate-risk group, when the CCRT-group was compared with the RT-group, as shown in Table 2 and Figure 2, CCRT was significantly superior in terms of recurrence rate (*P* = .01), PFS (log-rank; *P* = .0026), and OS (log-rank; *P* = .0435). Moreover, as shown in Table 3 and Figure 3, in the patients that displayed high-risk prognostic factors, the addition of concurrent nedaplatin-based chemotherapy also resulted in an improved outcome in terms of OS (log-rank; *P* = .0364). 

 In conclusion, our retrospective investigation demonstrated that the concurrent use of weekly nedaplatin with pelvic RT is safe and improves survival outcome in early-stage cervical cancer patients that display intermediate- or high-risk prognostic factors. Moreover, these results indicate that nedaplatin-based adjuvant CCRT can be considered as an alternative to cisplatin-based adjuvant CCRT.

## 3. Concurrent Nedaplatin-Based Chemoradiotherapy in Patients with FIGO Stage IIIb Cervical Cancer

### 3.1. Background

Radiotherapy is the major treatment modality for invasive cervical cancer and has achieved significantly improved treatment outcomes; however, substantial treatment failure still occurs, especially in advanced-stage patients [42].

One of the major clinical limitations in the management of advanced-stage disease is the size of the tumor. The tumor volume in patients with advanced-stage disease is usually large. It is generally accepted that the ability of radiotherapy to cure locally advanced cervical cancer is limited by the size of the tumor because the dose required to treat a large tumor exceeds the limit of toxicity in normal tissue [43]. Therefore, efforts to maximize local control and improve survival are necessary.

 For this purpose, various clinical studies have evaluated the survival benefit of adding concurrent chemotherapy. When added to pelvic RT, cisplatin was found to reduce the risk of death from cervical cancer by approximately 50% [25, 26, 44, 45]; however, its effects in patients with stage III or greater disease are far from optimal. According to a recent review of 18 clinical trials, the absolute 5-year survival increase was 10% (Stage IA-IIA), 7% (Stage IIb), and 3% (Stage III-IVa) [46]. Therefore, other therapeutic approaches must be tested in order to further improve outcomes.

 An important problem in the management of patients with advanced-stage cervical cancer is the presence of hydronephrosis as a result of ureteral obstruction caused by parametrial disease. It has previously been reported that the presence of hydronephrosis is an important indicator of a poor prognosis [47]. The precise incidence of ureteral obstruction is unknown, but it is reported to be 55.8% among patients with stage III-IV disease [47]. Although weekly cisplatin during RT has been reported to be well tolerated, its nephrotoxicity may limit its use for advanced-stage cervical cancer patients, especially in patients with impaired renal function due to ureteral obstruction. Therefore, the use of an agent such as nedaplatin that shows less nephrotoxicity as a radiosensitizer may improve the outcomes of these patients. 

 Based on the US National Cancer Institute (NCI) alert in 1999 [48], we have started the clinical use of nedaplatin-based CCRT using HDR-ICBT to determine if concurrent nedaplatin is a suitable alternative to cisplatin in patients with cervical cancer. 

 Recently, we conducted a retrospective analysis to evaluate whether nedaplatin-based CCRT is safe and superior to RT alone in Japanese patients with FIGO stage IIIb cervical cancer [20].

### 3.2. Clinical Findings

As shown in Table 5, 41 women with FIGO Stage IIIb cervical cancer were treated with either nedaplatin-based CCRT (*n* = 20) or RT alone (*n* = 21).

 Among the patients treated with CCRT, the median dose of nedaplatin administered was 35 mg/m^2^, and the median number of courses of nedaplatin was five. The optimal dose of concurrent weekly nedaplatin for patients with invasive cervical cancer treated by primary CCRT using EBRT and HDR-ICBT is still unknown, but has previously been investigated in several prospective clinical studies [14–17]. Of these, three studies recommended 30 mg/m^2^, and the other recommended 35 mg/m^2^ nedaplatin as a standard treatment regimen (Table 1). Therefore, the dose of nedaplatin utilized in our study was consistent with these previous studies.

 Overall, nedaplatin-based CCRT was well tolerated. All patients completed the planned EBRT and HDR-ICBT. As shown in Table 5, grade 3 or 4 acute toxicities were observed in 10 patients (50%) in the CCRT-group. The most frequently observed acute toxicity was neutropenia or a combination of neutropenia and thrombocytopenia. The overall incidence of grade 3-4 acute toxicities was significantly higher in the CCRT-group than in the RT-group. However, there were no significant differences in the duration of radiotherapy among these treatment groups (Table 5).

 With regard to radiotherapy using EBRT and LDR-ICBT, late toxicity from concurrent chemotherapy has been examined previously (Table 6), and no differences were found in the rates of bowel or bladder toxicity between the patients treated with RT alone and those treated with CCRT [49]. However, because of the lack of a controlled clinical study, it is not clear whether concurrent chemotherapy increases the incidence of late toxicity in the setting of radiotherapy using EBRT and HDR-ICBT. In our study, severe late complications were observed in two patients (10%) treated with CCRT. As shown in Table 5, the rate of severe late complications in the CCRT-group (10%) was similar to those of other series in which the patients had been treated with EBRT and HDR-ICBT without concurrent chemoradiotherapy [22–24], cisplatin-based CCRT using EBRT and LDR-ICBT [25, 26], or cisplatin-based CCRT using EBRT and HDR-ICBT [27–31]. Collectively, these results (including ours) suggest that the addition of concurrent weekly nedaplatin to EBRT and HDR-ICBT does not increase late toxicity.

**Figure 4:**
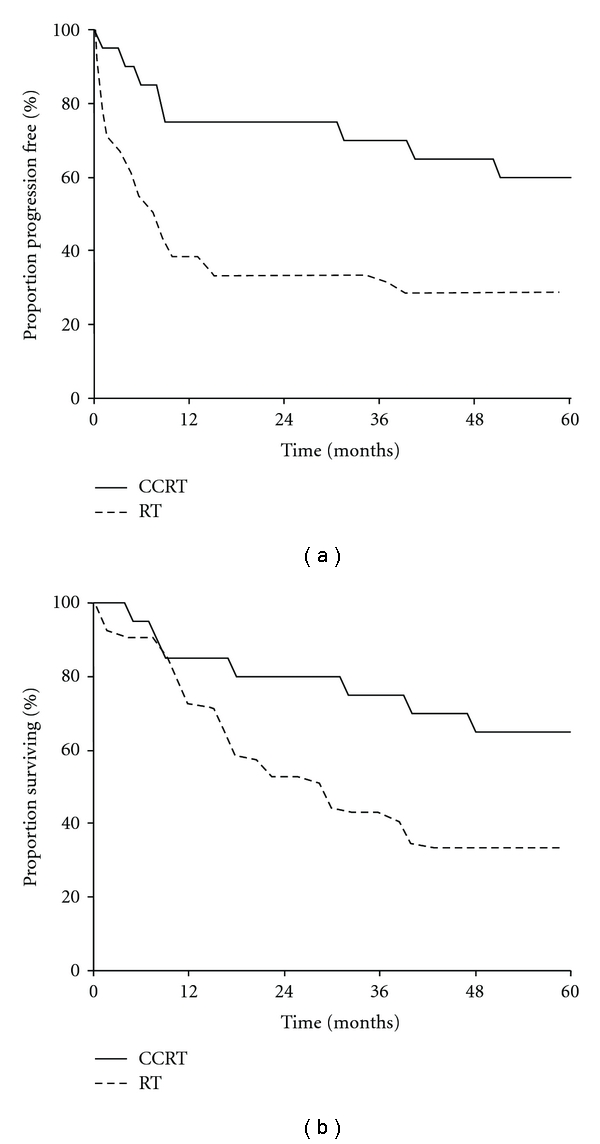
Progression-free survival (a) and overall survival (b) among patients in the CCRT- and RT-groups.

 When the CCRT-group was compared with the RT-group, as shown in Figure 4 and Table 5, CCRT was found to be significantly superior in terms of PFS (log-rank; *P* = .0015) and OS (log-rank; *P* = .0364), and the risk of death was decreased by 46% by the addition of nedaplatin-based concurrent chemotherapy. These results indicate that the addition of concurrent nedaplatin to pelvic EBRT plus HDR-ICBT significantly improved the prognosis of this patient population. The 5-year overall survival rate of 65% and the treatment failure rate of 35% found in our study are comparable to those demonstrated in previous clinical studies of cisplatin-based CCRT (Table 6). These results suggest that in this patient population nedaplatin can be considered as an alternative to cisplatin in the setting of chemoradiotherapy.

### 3.3. Future Directions and Prospects

Although the addition of concurrent nedaplatin-based chemotherapy to pelvic RT resulted in improved survival in our studies, a significant number of cervical cancer patients still suffered recurrence and died of their disease. Therefore, to further improve the prognosis of these patients, novel treatment strategies such as the use of nedaplatin-based combination chemotherapy as a radiosensitizer, the coadministration of nedaplatin with molecularly targeted agents, improved drug delivery strategies using delivery vehicles such as liposomes, or more conformal dose distributions with intensity-modulated RT need to be investigated in future trials.

 In contrast to cisplatin-based CCRT, due to the lack of prospective randomized studies, nedaplatin-based CCRT has not been convincingly proven to be clinically effective for the treatment of cervical cancer. However, given the advantage of patient's tolerance as well as its significant activity demonstrated in these studies, we believe that nedaplatin-based CCRT is a reasonable treatment option for this patient population. To definitively demonstrate the activity of nedaplatin-based CCRT, further investigation in a randomized controlled trial, for example, to compare concurrent nedaplatin versus cisplatin in the setting of CCRT, is warranted.

##  Conflict of Interests 

The authors declare that they have no conflicts of interest.

## Figures and Tables

**Table 1:** Summery of the literature on nedaplatin-based concurrent chemoradiotherapy.

Author (Reference)	Year	Study type	Setting	Stage	Results
J. Kodama [19]	2008	Phase I	Postoperative RT	Ib-IIb	Weekly 35 mg/m^2^ of nedaplatin was recommended.
Y. Kobayashi [21]	2009	Retro	Postoperative RT	Ib2-IIb	Biweekly 70 mg/m^2^ of nedaplatin was employed. CCRT was better than RT alone, but the improvement was not significant.
S. Mabuchi [20]	2009	Retro	Postoperative RT	IA2-IIb	Weekly 40 mg/m^2^ of nedaplatin was employed. CCRT was significantly better than RT alone.

Y. Onishi [14]	2002	Phase I	Definitive RT	III-IVa	Weekly 30 mg/m^2^ of nedaplatin was recommended.
K. Yoshinaga [15]	2007	Phase I	Definitive RT	Ib2-IIIb	Weekly 35 mg/m^2^ of nedaplatin was recommended.
Y. Yokoyama [16]	2008	Phase II	Definitive RT	Ib2-IVa	Weekly 30 mg/m^2^ of nedaplatin was employed.
Y. Niibe [17]	2008	Phase II	Definitive RT	IIIa-IVa	Weekly 30 mg/m^2^ of nedaplatin was employed.
S. Mabuchi [18]	2010	Retro	Definitive RT	IIIb	Weekly 35mg/m^2^ of nedaplatin was employed. CCRT was significantly better than RT alone.

CCRT: concurrent chemoradiotherapy, Retro: retrospective cohort study, RT: radiotherapy.

**Table 2:** Patient characteristics and treatment outcomes (Intermediate-risk patients).

			RT-group	CCRT-group	*P*-value
Patient characteristics	Number of patients		35	22	
	Age (mean)		49.8	49.6	
	Clinical stage	IA2	0	0	
		IB	25	18	
		IIA	10	4	
		IIB	0	0	
	Histology	Squamous cell	27	13	
		Adenocarcinoma	5	0	
		Adenosquamous	3	9	
		Others	0	0	

Treatment outcome	Duration of radiotherapy	Days	37	37	NS
	Patients with recurrence (%)		12 (34.3)	1 (4.5)	.01
	PFS (months)	Mean	29	36	.0026
	OS (months)	Mean	32.5	36	.0430

PFS: progression-free survival, OS: overall survival, CCRT: concurrent chemoradiotherapy, RT: radiotherapy.

**Table 3:** Patient characteristics and treatment outcomes (High-risk patients).

			RT-group	CCRT-group	*P*-value
Patient characteristics	Number of patients		34	34	
	Age (mean)		51.3	50.1	
	Clinical stage	IA2	0	0	
		IB	7	12	
		IIA	9	2	
		IIB	18	20	
	Histology	Squamous cell	21	25	
		Adenocarcinoma	13	8	
		Adenosquamous	0	1	
		Others	0	0	

Treatment outcome	Duration of radiotherapy	Days	38	38	NS
	Patients with recurrence (%)		16 (47.0)	9 (26.5)	NS
	PFS (months)	Mean	22.6	29.5	NS
	OS (months)	Mean	29.7	34.2	.0364

PFS: progression-free survival, OS: overall survival, CCRT: concurrent chemoradiotherapy, RT: radiotherapy.

**Table 4:** Grade 3-4 toxicities (acute and late toxicities).

			CCRT-group	RT-group
			(*n* = 56)	(*n* = 69)
Acute	Number of patients with Grade 3-4 acute toxicity (%)		36 (64.3)	8 (11.6)
	Hematologic	Neutropenia	30	4
		Thrombocytopenia	3	0
		Neutropenia + Thrombocytopenia	3	1
	Nonhematologic	Bowel obstruction	1	1
		Diarrhea	1	2
		Dermatitis	1	0

Late	Number of patients with Grade 3-4 late toxicity (%)		0	1 (1.8)
		Vesicovaginal fistula	0	1

RT: radiotherapy, CCRT: concurrent chemoradiotherapy.

**Table 5:** Patient characteristics and treatment outcomes.

			RT-group	CCRT-group	*P*-value
Patient characteristics	Number of patients		21	20	
	Median age		67	59	
	Histology	SCC	21	20	
		Others	0	0	
	Dose of nedaplatin administered, mg/m^2^	Median (range)	—	35 (10–45)	
	Courses of nedaplatin administered	Median (range)	—	5 (2–5)	

Treatment outcome	PFS, months	Median (range)	7 (0–60)	60 (0–60)	.0015
		Mean	21.4	43.3	
	OS, months	Median	29 (1–60)	60 (5–60)	.0364
		Mean	32.3	47	
	Duration of radiotherapy	Median (range)	47 (41-61)	45 (40–57)	NS
	Patients with recurrence (%)		14 (66.7%)	7 (35%)	NS
	Patients with grade 3-4 acute toxicity (%)		1 (4.8%)	10 (50%)	.0014
	Patients with grade 3-4 late toxicity (%)		0 (0%)	2 (10%)	NS

PFS: progression-free survival, OS: overall survival, SCC: squamous cell carcinoma, CCR: concurrent chemoradiotherapy, RT: radiotherapy.

**Table 6:** Literature review: survival, treatment failure, and complications.

Author (Reference)	Year	Study type	Concurrent chemotherapy	Brachytherapy	Stage	Followup	Overall Survival (%)	Treatment Failure (%)	Late toxicity (grade 3-4) (%)
									
T. Teshima [22]	1993	RCT	—	HDR	III	5 years	53	47	10
M. Hareyama [23]	2002	RCT	—	HDR	III	5 years	69	49	7
P. Lertsanguansinchai [24]	2004	RCT	—	HDR	IIIb	3 years	71	30	7

M. Morris [25]	1999	RCT	Cisplatin	LDR	III-IVa	5 years	63	42	12
P. G. Rose [26]	1999	RCT	Cisplatin	LDR	II-IVa	4 years	66	38	1.7
T. Toita [27]	2005	Retro	Cisplatin	HDR	I-III	3 years	79	33	2
Y. L. Chung [28]	2005	Phase I/II	Cisplatin	HDR	IIb-IVa	3 years	83	19	6
S. W. Chen [29]	2006	Retro	Cisplatin	HDR	IIb-III	4 years	74	46	14
R. Potter [30]	2006	Retro	Cisplatin	HDR	Ib-IVa	3 years	61	44	4
P. Novetsky [31]	2007	Retro	Cisplatin	HDR	III-IV	5 years	65	35	6
S. Mabuchi [20]	2010	Retro	Nedaplatin	HDR	IIIb	5 years	65	35	10

RCT: randomized controlled study, Retro: retrospective study, HDR: high-dose rate brachytherapy, LDR: low-dose rate brachytherapy.

## Abbreviations

OS: Overall survival
PFS: Progression-free interval
ICBT: Intracavitary brachytherapy
HDR: High dose rate
LDR: Low dose rate
EBRT: External beam radiotherapy
RT: Radiotherapy
CCRT: Concurrent chemoradiotherapy.
